# Implementation of an intravenous sotalol initiation protocol: Implications for feasibility, safety, and length of stay

**DOI:** 10.1111/jce.15819

**Published:** 2023-02-22

**Authors:** Albert Y. Liu, Jessica Charron, Dana Fugaro, Scott Spoolstra, Rachel Kaplan, Graham Lohrmann, Xu Gao, Hawkins Gay, Rod Passman, Susan Kim, Albert C. Lin, Alexandru Chicos, Rishi Arora, Kaustubha Patil, Anna Pfenniger, Bradley P. Knight, Nishant Verma

**Affiliations:** 1 Division of Cardiology, Northwestern University, Chicago, Illinois, USA; 2 Division of Cardiology, Bluhm Cardiovascular Institute, Northwestern Memorial Hospital, Chicago, Illinois, USA; 3 Division of Cardiology, Medical University of South Carolina, Charleston, South Carolina, USA

**Keywords:** antiarrhythmic drugs, atrial fibrillation, atrial flutter, intravenous sotalol, premature ventricular contractions, quality improvement, ventricular tachycardia

## Abstract

**Introduction:**

Oral sotalol initiation requires a multiple-day, inpatient admission to monitor for QT prolongation during loading. A 1-day intravenous (IV) sotalol loading protocol was approved by the United States Food and Drug Administration in March 2020, but limited data on clinical use and administration currently exists. This study describes implementation of an IV sotalol protocol within an integrated health system, provides initial efficacy and safety outcomes, and examines length of stay (LOS) compared with oral sotalol initiation.

**Methods:**

IV sotalol was administered according to a prespecified initiation protocol to adult patients with refractory atrial or ventricular arrhythmias. Baseline characteristics, safety and feasibility outcomes, and LOS were compared with patients receiving oral sotalol over a similar time period.

**Results:**

From January 2021 to June 2022, a total of 29 patients (average age 66.0 ± 8.6 years, 27.6% women) underwent IV sotalol load and 20 patients (average age 60.4 ± 13.9 years, 65.0% women) underwent oral sotalol load. The load was successfully completed in 22/29 (75.9%) patients receiving IV sotalol and 20/20 (100%) of patients receiving oral sotalol, although 7/20 of the oral sotalol patients (35.0%) required dose reduction. Adverse events interrupting IV sotalol infusion included bradycardia (seven patients, 24.1%) and QT prolongation (three patients, 10.3%). No patients receiving IV or oral sotalol developed sustained ventricular arrhythmias before discharge. LOS for patients completing IV load was 2.6 days shorter (mean 1.0 vs. 3.6, *p* < .001) compared with LOS with oral load.

**Conclusion:**

IV sotalol loading has a safety profile that is similar to oral sotalol. It significantly shortens hospital LOS, potentially leading to large cost savings.

## 1 | INTRODUCTION

Atrial fibrillation (AF) is the most common rhythm disturbance, with lifetime risk estimates between 20% and 30% in the United States.^1^ The disease is complex and often difficult to treat for clinicians. It represents a considerable cost burden with an estimated incremental cost of $26 billion in 2010.^2^ Antiarrhythmic drugs (AADs) remain a cornerstone of therapy for management of AF. The Class III AAD sotalol has QT-prolonging effects that necessitate multiple days of inpatient hospitalization for observation during oral loading. This has been estimated to cost more than $10 000 per patient for a standard 3-day admission, with the greatest proportion of cost coming from room and board.^3^ The use of inpatient beds for these drug loads can also significantly impact hospital capacity to accept and manage other patients, given staffing and bed shortages.^4^

Intravenous (IV) sotalol was first approved for use by the United States Food and Drug Administration (FDA) as a substitute for oral therapy in patients with supraventricular arrhythmias, life-threatening ventricular arrhythmias, and AF/flutter, but with recommendations for infusion over 5 h.^5^ InMarch 2020, the IV formulation of the drug received FDA approval for the use in expedited loading of oral sotalol.^6^ This approval was based on the Model-Informed Drug Development regulatory path, with simulated data showing that an initial 1 h loading dose of IV sotalol followed by two oral doses in 24 h reflected maximum QT prolongation over a 1-day observation period.^7^ The newly approved use case can reduce loading time by 2 days or more, with even greater reductions for those with impaired renal function. Despite the higher drug cost of IV sotalol, the anticipated reduction in hospital length of stay (LOS) with IV sotalol is expected to lead to significant cost savings when compared with oral sotalol initiation.^3^

Thus far, available data supporting the use of 1-day IV sotalol initiation has been limited to model-informed simulation data.^7^ A single-center experience with a 1-day sotalol loading protocol for atrial arrhythmias has been briefly described, but there are no studies comparing IV versus oral sotalol load in a clinical context.^8^ This study aims to describe implementation of a 1-day IV sotalol initiation protocol for atrial and ventricular arrhythmias at a learning health system and to provide initial feasibility and safety outcomes compared with oral sotalol initiation.

## 2 | METHODS

### 2.1 | Patient selection and protocol description

Administration of IV sotalol began in August 2021 using the protocol described in the Supporting Information: Appendix. Adult patients with a creatinine clearance (CrCl) > 30 ml/min and a primary indication of atrial or ventricular arrhythmias were included. Patients with bradycardia <60 b.p.m. or corrected QT (QTc) interval >450ms were excluded, except in select cases with the approval and oversight of an attending cardiac electrophysiologist. Those with a paced QRS or bundle branch block were permitted a QTc interval up to 500ms. Patients with acute decompensated heart failure or cardiogenic shock were also excluded, although there was no prespecified lower limit for left ventricular ejection fraction (LVEF) or blood pressure. Patients had their baseline electrolytes drawn and repleted, and a baseline electrocardiogram (ECG) was obtained. If they presented in a sustained atrial arrhythmia, cardioversion to sinus rhythm was performed before infusion. IV sotalol was then infused over 60min with one-to-one nursing, frequent vital signs, and continuous cardiac telemetry. If the heart rate dropped below 60 b.p.m., the QT interval was used instead of the QTc interval for decision making. ECGs were obtained every 15min during infusion. The infusion was stopped if the patient developed bradycardia <50 b.p.m. or had prolongation of the QT/QTc interval to greater than 500 ms (550 ms for those with paced QRS or bundle branch block). After completion of infusion, patients were admitted to the inpatient telemetry unit for the remaining two oral doses, with subsequent ECGs 2–4 h after each oral dose.

Patients undergoing oral sotalol initiation for atrial and ventricular arrhythmias from January 2021 to June 2022 were included for comparison with IV sotalol. Inclusion and exclusion criteria were identical. Oral doses were given based on standard prescribing regimens using CrCl, with ECGs similarly performed 2–4 h after each dose of sotalol. Patients were monitored for five oral doses before discharge. Patients with sustained atrial arrhythmias were cardioverted after the fifth oral dose of sotalol and monitored for an additional day after cardioversion. Those who experienced bradycardia <50 b.p.m. or QT/QTc prolongation >500 ms (550 ms for those with paced QRS or bundle branch block) either had their oral sotalol dose reduced or discontinued.

### 2.2 | Demographics and clinical assessment

Patient demographics and clinical characteristics included age, sex, indication for sotalol initiation, prescription of atrioventricular (AV) nodal blocking agents before sotalol initiation, CrCl, and LVEF. Patient clinical data were manually extracted via chart review. Heart rates and QT/QTc intervals by ECG were obtained every 15 min during sotalol infusion and 2–4 h after each oral sotalol dose. The QT interval was used for decision making when the heart rate was below 60 b.p.m. Abnormal QT/QTc intervals during sotalol loading were verified with manual calculation using the Bazett formula by the inpatient electrophysiology team. Heart rates and QT/QTc intervals were subsequently adjudicated by a study investigator.

### 2.3 | Safety endpoints and outcome measures

Outcomes related to sotalol dosing, changes in heart rate and QT/QTc interval in response to sotalol infusion and oral sotalol doses, and LOS were assessed. Safety outcomes included the development of sustained ventricular tachycardia or ventricular fibrillation, bradycardia <50 b.p.m., acute decompensated heart failure, hypotension requiring vasopressors, and QT/QTc prolongation >500 ms (or 550 ms for those with paced QRS or bundle branch block). All events were determined by review of electronic medical records, with heart rates and QT/QTc intervals determined by evaluation of ECGs obtained during clinical care.

### 2.4 | Statistical analysis

Baseline characteristics and outcome comparisons between oral and IV sotalol initiation cohorts were performed using the Student’s *t* test for noncategorical variables and with the χ^2^ test for categorical variables. The one-sample Sign test was used to compare LOS given the non-normal distribution.

## 3 | RESULTS

### 3.1 | Demographics and clinical characteristics

Between January 1, 2021 and June 30, 2022, a total of 29 patients underwent IV sotalol initiation and 20 patients underwent oral sotalol initiation. Baseline demographics and clinical characteristics for both groups are reported in Table 1. The average age of the IV sotalol initiation group was 66.0 years and 27.6% were female. The primary indication for IV loading was 75.9% atrial arrhythmias and 24.1% ventricular arrhythmias. Average CrCl was 90.6 ml/min. LVEF ranged from 31% to 69% and average LVEF was 52.8%. For the oral sotalol initiation group, average age was 60.4 years and 65.0% were female. Primary indication for oral loading was 55.0% atrial arrhythmias and 45.0% ventricular arrhythmias. Average CrCl was 99.5 ml/min. LVEF ranged from 26% to 75% and average LVEF was 54.8%.

Almost all patients undergoing IV and oral sotalol initiation were on AV nodal blocking agents (96.6% and 95.0%, respectively) before presentation. The distribution of specific classes of agents is listed in Table 1. The total daily metoprolol milligram equivalent dose averaged 80 mg in the IV sotalol group and 76 mg in the oral sotalol group. The total daily diltiazem milligram equivalent dose averaged 135 mg in the IV sotalol group and 240 mg in the oral sotalol group. Only 6 of 29 patients (20.7%) in the IV sotalol group and 7 of 20 patients (35.0%) in the oral sotalol had these agents held or reduced immediately before sotalol initiation.

### 3.2 | Clinical and safety outcomes

**FIGURE 1:**
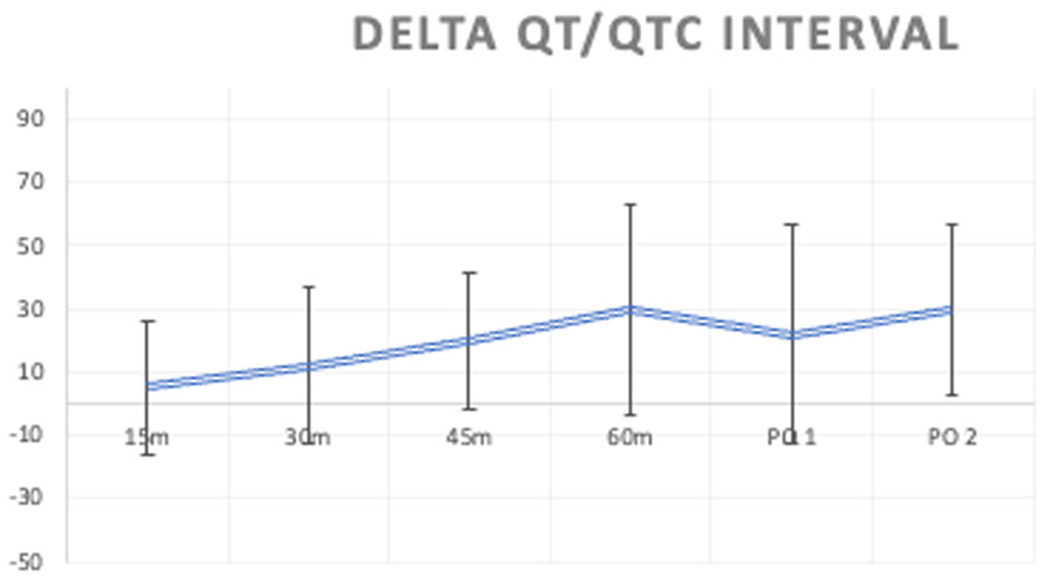
Mean change from baseline of the QT/QTc interval (ms) during IV sotalol initiation at 15, 30, 45, and 60 min into the infusion, as well as after the first and second oral dose. Error bars represent SDs.

Changes in heart rate and QT/QTc interval over the course of IV sotalol initiation are described in Table 2. At the end of the 60 min IV sotalol infusion, the mean QT/QTc interval had increased from baseline by 29.7 ms; at discharge after two additional oral doses, the QT/QTc interval had increased from baseline by 29.8 ms (Figure 1).

Of the 29 patients undergoing IV sotalol initiation, 7 had discontinuation of the infusion due to significant bradycardia (Table 3). Six out of seven of these patients were on AV nodal blocking agents (five on metoprolol and one on diltiazem) that were not discontinued or dose reduced before the start of IV sotalol. None of the patients who developed significant bradycardia required transcutaneous pacing or IV chronotropic support. Conversion to oral sotalol load was attempted for two of these patients but ultimately aborted due to persistent bradycardia. One patient received 45 min of the IV sotalol infusion before discontinuation for bradycardia and was discharged after two additional oral doses. The other four patients were discharged off sotalol without attempts at further oral loading. Of the seven patients who developed significant bradycardia, three also had prolongation of the QT interval that would have required discontinuation. A total of 22 out of 29 patients (75.9%) underwent successful IV sotalol initiation. Of these, 10 of 22 patients (45.5%) were discharged on the 80 mg dose of sotalol and 12 of 22 patients (54.5%) were discharged on the 120 mg dose of sotalol.

All patients undergoing oral sotalol initiation were able to complete their sotalol load, but seven required dose reductions due to bradycardia and/or QT prolongation. The two patients who required dose reduction due to bradycardia did not have their AV nodal blocking agents held before oral sotalol load. Seven of 20 patients (35.0%) were discharged on the 80 mg dose of sotalol and 13 of 20 patients (65.0%) were discharged on the 120 mg dose of sotalol. Four patients required cardioversion to sinus rhythm at the end of oral load.

There were no clinically significant increases in premature ventricular contraction burden noted in either group after oral or IV sotalol initiation. No patients developed hypotension requiring vasopressors, acute decompensated heart failure, or sustained ventricular arrhythmias. Average LOS for the IV sotalol initiation group was 1 day compared with 3.6 days in the oral sotalol group (*p* < .001).

## 4 | DISCUSSION

In this study, IV sotalol was found to be a safe and well tolerated method of sotalol initiation for both atrial and ventricular arrhythmias. Almost one in four patients undergoing IV sotalol initiation had serious bradycardia, but six/seven of them were on AV nodal blocking agents at the time of initiation. Discontinuing or dose reducing these agents before IV sotalol initiation may help to mitigate these effects. QT prolongation was noted only in the context of serious bradycardia in our IV sotalol cohort, with a rate of ~10% matching those found in other real-world data.^8^ No patients undergoing oral sotalol initiation had the drug discontinued due to bradycardia or QT prolongation, but 35% of the patients had doses reduced to less efficacious doses.^9^ No patients in either group had life threatening sustained ventricular arrhythmias during their hospitalization. Most notably, patients undergoing IV sotalol initiation had a significantly shorter hospital LOS than patients undergoing oral sotalol initiation, with a mean difference of 2.6 days. Based on cost estimates done by Varela et al.,^3^ this represents potential savings of more than $4000 per patient.

Additional analysis was performed evaluating changes in the QT/QTc interval during IV sotalol infusion and after the first and second oral dose. The increase in the QT/QTc interval by the end of the IV infusion approximates that seen at discharge. These findings provide preliminary clinical corroboration for pharmacokinetics–pharmacodynamic models that simulate peak serum concentration of sotalol immediately after infusion.^7^ Taken together, this suggests that discharge after infusion may be safe from a QT prolongation perspective. Efforts to decrease IV sotalol initiation time are already underway, with the DASH-AF study looking at discharge after IV infusion and a single oral sotalol dose currently enrolling patients.^10^

### 4.1 | Study limitations

Because of the small cohort size, conclusions about changes in the QT/QTc interval during and after infusion are tempered by large intra- and inter-patient variability in the QT/QTc interval. The PEAKS registry is a multicenter registry currently being established of patients receiving IV sotalol initiation that will further refine understanding of QT prolongation during and after infusion, with both clinical and pharmacokinetic–pharmacodynamic data.^11^ This study’s cohort also tended to be healthier, with overall preserved kidney function and low normal LVEF, and data may not be as generalizable to those with decreased renal clearance or more significantly depressed ejection fractions for whom sotalol can be more dangerous. There were also significantly more women in the oral sotalol load group, which may bias QT interval comparisons given the known differences in repolarization between genders. Lastly, patients were only followed until discharge and longer-term safety outcomes are not available. These longer-term outcomes are presumed to be similar to patients loaded with oral sotalol.

### 4.2 | Conclusion

The use of a 1-day IV sotalol loading protocol for atrial and ventricular arrhythmias is safe and well tolerated. It reduces LOS from an average of 3.6 days to 1 day compared with oral sotalol loading, which can translate into significant cost and resource savings. QT prolongation appears to be near its greatest immediately after sotalol infusion, which suggests discharge after infusion may be possible. Studies evaluating accelerated discharge after a single oral dose as well as an expanded multicenter registry of patients undergoing IV sotalol initiation are forthcoming.

## Supplementary Material

Supplemental_File

## Figures and Tables

**TABLE 1:** Baseline demographics and clinical characteristics of patients receiving IV and oral sotalol initiation.

	IV sotalol	Oral sotalol	*p*

Patients (*n*)	29	20	–

Age (years)	66.0 ± 8.6	60.4 ± 13.9	.087

Sex (*n*, % female)	8 (27.6%)	13 (65.0%)	.009

Indication (n, %)			.168
- AF	17 (58.6%)	11 (55.0%)	
- AFL	2 (6.9%)	0	
- AT	3 (10.3%)	0	
- PVC/VT	7 (24.1%)	9 (45.0%)	

CrCl (ml/min)	90.6 ± 24.1	99.5 ± 42.6	.360

LVEF (%)	52.8 ± 12.0	54.8 ± 12.9	.589

AVN blocking agents (*n*, %)^a^			.681
- BB	27 (93.1%)	19 (95.0%)	
- CCB	2 (6.9%)	1 (5.0%)	
- Digoxin	2 (6.9%)	0	
- None	1 (3.4%)	1 (5.0%)	

Abbreviations: AF, atrial fibrillation; AFL, atrial flutter; AT, atrial tachycardia; AVN, atrioventricular nodal; BB, β-blockers; CCB, calcium channel blockers; CrCl, creatinine clearance; IV, intravenous; LVEF, left ventricular ejection fraction; PVT/VT, premature ventricular contraction/ventricular tachycardia.

a Percentages do not add up to 100% as some patients were on combination therapy.

**TABLE 2:** Changes in HR and QT/QTc interval during IV sotalol initiation.

Patients completing initiation (*n* = 22)	Baseline (before receiving sotalol)	At end of IV sotalol infusion	At discharge (after receiving two oral doses)
HR (mean ± SD)	71.2 ± 11.1	66.5 ± 13.8	61.8 ± 9.9
QTc (mean ± SD)	456.9 ± 39.2	486.6 ± 35.1	486.7 ± 28.7

Abbreviations: HR, heart rate; IV, intravenous.

**TABLE 3:** Clinical and safety outcomes during hospitalization for IV and oral sotalol initiation.

	IV Sotalol	Oral sotalol	*p*-value
Bradycardia leading to drug discontinuation or dose reduction (*n*, %)	7 (24.1%)	2 (10.0%)	.209
QT prolongation leading to drug discontinuation or dose reduction (*n*, %)	3 (10.3%)	6 (30.0%)	.081
VT/VF	0	0	–
Average LOS (mean, range)	1.0 (1–1)	3.6 (3–6)	<.001

Abbreviations: IV, intravenous; LOS, length of stay; VT/VF, ventricular tachycardia/ventricular fibrillation.
