# Supplementary material for: Implementation of an intravenous sotalol initiation protocol: Implications for feasibility, safety, and length of stay
Source: J Cardiovasc Electrophysiol. Author manuscript; Available in PMC 2023 Dec 6. (PMC10699543; doi:10.1111/jce.15819)
Supplement: Supplemental_File [file NIHMS1946511-supplement-Supplemental_File.docx]

*Supplemental Appendix*

**IV Sotalol Infusion Clinical Protocol**

Logistics

- Planned day of admission: Tuesday
- Estimated volume: one patient a week
- Plan to present in the electrophysiology (EP) peri-operative holding area, the Cardiac Recovery and Observation Unit (CROU), with subsequent admission to telemetry bed (hospitalist or cardiology floor service)
- EP consult service (general or EP fellow) to write short IV sotalol infusion consult note at time of admission: will include oral dosing and timing interval

Pre-admission EMR order set (to be auto-ordered by CROU nurse or placed by primary EP physician / EP clinic nurses) – complete details in protocol below

1. Prior to presenting to CROU, to be done in outpatient lab if possible
   1. ECG
   2. Basic metabolic panel and magnesium level
   3. COVID test
2. While in CROU
   1. Vital signs on arrival
   2. Baseline ECG and labs if not obtained prior; note baseline QTc* interval
   3. Replete K > 4, Mg > 2 (ideally, automated repletion order set that nurses can use) based on morning labs
   4. Direct current cardioversion (DCCV) if in atrial fibrillation
   5. ECG post-DCCV
   6. IV sotalol dose to be ordered by consult EP service after creatinine and ECG have resulted
   7. IV sotalol infusion over 1 hour (doses noted in Table 1 below)
   8. Vital signs every 15 minutes during sotalol infusion
   9. Print telemetry strips (lead II) and obtain ECG every 15 minutes during sotalol infusion
   10. ECG 2 hours after infusion
   11. Vital signs per unit protocol after infusion – maintain continuous telemetry

Floor Team Orders (after Step 9 in the outpatient protocol below)

1. PO sotalol (dose and timing described in Table 1 and “Protocol Timing” sections below
2. ECGs 2-4 hours post-sotalol (as described in “Protocol Timing” below)
3. Admitting team to read consult note and direct any follow-up questions to EP consult pager

**Outpatient Protocol**

1. Present to CROU for IV Sotalol load with continuous telemetry
2. Obtain baseline vital signs, and nurse to review ECG to assess rhythm and ensure QTc <= 450 ms. To discuss with EP consult pager if necessary.
3. Replete K > 4, Mg > 2. No need to repeat labs afterwards prior to starting IV sotalol unless K < 3, Mg < 1.2.
4. Calculate CrCl using Cockcroft-Gault formula; CrCl must be above 30 to proceed
   1. For patients weighing > 40% above their ideal body weight, use adjusted body weight to determine CrCl:
5. If the patient is in atrial flutter or atrial fibrillation, proceed with DCCV per usual protocol
   1. If patient has immediate return of AF, discuss with EP Consult Pager
6. Repeat ECG in normal sinus rhythm to ensure QTc <= 450 ms. Note this value as baseline QTc.
7. CROU nurse to administer IV sotalol over one hour in the CROU room.
   1. Vital signs and QTc* will be measured every 15 minutes. Print telemetry strip and obtain ECG every 15 minutes for QTc measurement.
   2. CROU nurse to review QTc on ECG, to page EP Consult Pager to review if unclear or QTc > 500
   3. If HR decreases to below 50, then call EP consult pager for further guidance
   4. If QTc prolongs to > 500 ms or increases by more than 20% from baseline, then:
      1. If loading for 80 mg, discontinue sotalol and discuss next steps with EP Consult Pager
      2. If loading for 120 mg:
         1. If CrCl >= 60, hold sotalol for 24 hours and resume IV sotalol at lower dose for 80 mg load
         2. If CrCl < 60, hold sotalol and discharge with plan for another rhythm control strategy or to re-admit at least 72 hours later for repeat IV sotalol for 80 mg load
   5. CROU nurse to note time of completion of infusion
8. CROU nurse to coordinate admission to telemetry bed (hospitalist or cardiology floor service); page EP consult pager with questions
9. Vitals can be obtained per typical CROU protocol; please maintain patient on continuous telemetry
10. Obtain QTc via ECG 2-4 post infusion
11. First oral dose of sotalol to be given (*See below for timing of oral dosing depending on CrCl*)
12. Obtain QTc 2-4 hours after administration of first oral dose
13. Order basic metabolic panel and magnesium level in the morning
    1. Replete K>4, Mg >2
    2. Adjust dose for CrCl if necessary, please notify EP consult pager if doing so (*see below*)
14. Second oral dose to be given
15. Obtain QTc 2-4 hours after administration of second oral dose
16. Discharge if CrCl between >=30
17. Post-discharge ECG in 2-3 days (included in initial data set)

*QTc during infusion will be measured both by ECG and by telemetry. QT interval to be used if HR drops below 60.

**Table 1. Timing and Dose of intravenous and oral sotalol load**

| **CrCl (mL/min)** | **IV sotalol dose if the planned oral dose is 80mg*^** | **IV sotalol dose if the planned oral dose is 120 mg^** | **Minimum delay to first oral dose** | **Oral dosing interval** |
| --- | --- | --- | --- | --- |
| >90 | 60 mg | 90 mg | 4 hours | 12 hours |
| 60-90 | 82.5 mg | 125 mg | 4 hours | 12 hours |
| 30-60 | 75 mg | 112.5 mg | 6 hours | 24 hours |
| 10-30 | 75 mg | 112.5 mg | 12 hours | 48 hours |

Protocol Timing for CrCl > 60

- 630 AM: Check in
- 630 AM – 11 AM: Check baseline ECG, COVID swab, replete K and Mg as necessary (no need to recheck labs after repletion), cardioversion
- 11 AM - 12 PM: IV sotalol infusion
- 4 PM: First oral dose given (4 hours after completion of IV sotalol load)
- 6 PM: Obtain ECG and review QTc*
  - Reach out to EP Consult Pager if questions or if QTc > 500 or more than 20% from baseline)
  - If receiving 80 mg of oral sotalol, hold sotalol and discuss next steps with EP Consult Team
  - If receiving 120 mg of oral sotalol, down-titrate to 80 mg at next dose in consultation with EP Consult Team
- Day 2, 4 AM: Second oral dose given (12 hours after first oral dose)
  - If CrCl fallen to 30-60, please halve the second dose
  - If CrCl fallen to < 30, hold and contact EP Consult Pager
- Day 2, 6 AM: Obtain ECG and review QTc
  - If QTc < 500 or < 20% increase from baseline, discharge patient with maintenance dosing per Table 1
  - If QTc > 500 ms or more than 20% from baseline, please reach out to EP Consult Pager
- Day 2, 7 AM: Discharge patient

Protocol Timing for CrCl 30-60

- 630 AM: Check-in
- 630 AM – 11 AM: Check baseline ECG, replete K and Mg as necessary (no need to recheck labs after repletion), cardioversion
- 11 AM – 12 PM IV sotalol infusion
- After 12 PM: Transfer patient to medicine floor bed
- 6 PM: First oral dose given (6 hours after completion of IV sotalol load)
- 8 PM: Obtain ECG and review QTc
  - Reach out to EP pager if questions or if QTc > 500 or more than 20% from baseline)
    - If receiving 80 mg of oral sotalol, hold sotalol and discuss next steps with EP Consult Team
    - If receiving 120 mg of oral sotalol, down-titrate to 80 mg at next dose in consultation with EP Consult Team
- Day 2, **6 PM**: Second oral dose given (24 hours after first oral dose)
  - If CrCl fallen to < 30, hold and contact EP Consult Pager
- Day 2, 8 PM: Obtain ECG and review QTc
  - If QTc < 500 or < 20% increase from baseline, discharge patient with maintenance dosing per Table 1
  - If QTc > 500 ms or more than 20% from baseline, please reach out to EP Consult Pager
- Day 2, 9 PM: Discharge patient

*QTc during infusion will be measured both by ECG and by telemetry. QT interval to be used if HR drops below 60.
